# Will Climate Change Affect Outbreak Patterns of Planthoppers in Bangladesh?

**DOI:** 10.1371/journal.pone.0091678

**Published:** 2014-03-11

**Authors:** M. P. Ali, Dingcheng Huang, G. Nachman, Nur Ahmed, Mahfuz Ara Begum, M. F. Rabbi

**Affiliations:** 1 Entomology Division, Bangladesh Rice Research Institute, Gazipur, Bangladesh; 2 International Affairs Office, University of Chinese Academy of Sciences, Beijing, China; 3 Department of Biology, University of Copenhagen, Copenhagen, Denmark; The Ohio State University, United States of America

## Abstract

Recently, planthoppers outbreaks have intensified across Asia resulting in heavy rice yield losses. The problem has been widely reported as being induced by insecticides while other factors such as global warming that could be potential drivers have been neglected. Here, we speculate that global warming may increase outbreak risk of brown planthopper (*Nilaparvata lugens* Stål.). We present data that demonstrate the relationship between climate variables (air temperature and precipitation) and the abundance of brown planthopper (BPH) during 1998–2007. Data show that BPH has become significantly more abundant in April over the 10-year period, but our data do not indicate that this is due to a change in climate, as no significant time trends in temperature and precipitation could be demonstrated. The abundance of BPH varied considerably between months within a year which is attributed to seasonal factors, including the availability of suitable host plants. On the other hand, the variation within months is attributed to fluctuations in monthly temperature and precipitation among years. The effects of these weather variables on BPH abundance were analyzed statistically by a general linear model. The statistical model shows that the expected effect of increasing temperatures is ambiguous and interacts with the amount of rainfall. According to the model, months or areas characterized by a climate that is either cold and dry or hot and wet are likely to experience higher levels of BPH due to climate change, whereas other combinations of temperature and rainfall may reduce the abundance of BPH. The analysis indicates that global warming may have contributed to the recent outbreaks of BPH in some rice growing areas of Asia, and that the severity of such outbreaks is likely to increase if climate change exaggerates. Our study highlights the need to consider climate change when designing strategies to manage planthoppers outbreaks.

## Introduction

Outbreak frequency of *Nilaparvata lugens* (brown planthopper; BPH) has been increasing in Asian rice growing countries in recent years (2005–2012) [1]. These trends are widely linked to adverse effects on BPH natural enemies of the increased use of broad spectrum insecticides for control of a range of pests [2]–[11]. They are rarely linked to environmental changes such as global warming that can directly or indirectly influence outbreak trends in rice growing regions [12]. Insecticide resistance is another reason for the increase.

Rice is extremely important for human food supply, yet a comprehensive understanding of how climate change affects rice pests is still premature. As the global climate continues to change, there is a need to understand how planthoppers respond to low-frequency (interannual or longer period) climatic variability. Such low frequency climatic variability is of interest because of its significance for the understanding and prediction of protracted climatic anomalies [13]. Since ongoing climate change has a profound effect on insect pests and changes their pest status [14] and population dynamics [15], climate change probably contributes to recent destructive outbreaks of brown planthopper (BPH) *Nilaparvata lugens* (Stål) (Homoptera: Delphacidae). BPH is one of the most serious pests of rice in both temperate and tropical regions of East and South Asia and has become especially problematic over the past few years [16]. They are active throughout the year in the tropics [17]. In Bangladesh, where rice is grown year-round, they are prevalent throughout the year and can produce 8–11 generations per a year [personal communication, Nur Ahmed].

BPH’s life cycle comprises three distinct stages: egg, nymph and adult. Both nymphs and adults directly damage rice plants through sucking the cell sap from the base (stem) of the plants [18] and by transmitting viruses such as rice ragged stunt (RRSV) and rice grassy stunt (RGSV) [19]–[20] which cause severe losses. In 2005–2006, more than 485,000 hectares of rice in southern Vietnam were severely affected by viral diseases seemingly spread by BPH, resulting in the loss of 828,000 tons of rice valued at US$120 million [21].

Recently, outbreaks of BPH have become ever more common in Asia where farmers experience devastating losses [1], and have threatened rice production in parts of Thailand, Philippines, Indonesia, India, Bangladesh, Malaysia and China in the years of 2009–2010. Global warming may have been a contributing factor because steadily warmer autumns have occurred since the 1990s. Changed climatic conditions of particular interest are milder winters and warmer summers as well as changing patterns of precipitation. The latter includes increased risk of both extreme precipitation and severe dry spells. These conditions are predicted by the IPCC [22] to become more frequent due to changes in the global climate. Overall, temperature is predicted to increase 1.5–4.5°C during the present century [23] and precipitation to rise 10 to 15% because a warmer atmosphere holds more water [24]–[25]. These changes could profoundly affect the population dynamics and the status of insect pests of crops [24], [26]. At the same time, migrant species may also change status to become major rice pests. Thus, in Japan *N. lugens* was dominant until 1984, *Sogotula furcifera* by 1993 and *Cnaphalocrosis medinalis* up to now [27].

Reported BPH outbreaks are mostly attributed to the application of insecticides in 2008–2012 [2]–[11]. Other factors that may induce the problem and threaten rice production in major rice producing countries are greatly neglected although temperature has already started to rise with concurrent changes in precipitation patterns. For an array of insects these phenomena may affect e.g. phenology and pest status. Analysis of trapping data from several decades indicates that climate change also causes a change in the phenology of insects [28]. It is suggested that the use of the lower developmental threshold (*T*_0_) and the thermal constant (*K*) that have been reported for insects would be useful in predicting the phenology of insect communities under global warming [29]. Therefore, a more detailed analysis of correlations between climatic parameters and phenology, abundance and pest status are needed**.** The hypothesis is that a higher frequency of destructive outbreaks of plant hoppers is related to climatic changes.

In this paper, we use concurrent information on air temperatures, precipitation and abundance of BPH, recorded in Bangladesh over a 10-years period, to analyze the complex relationship between the climate variables and the dynamics of BPH. The aim of the present study is to provide a better understanding of the role of global warming in eliciting destructive outbreaks of BPH during the recent years as well as predicting its future role.

## Materials and Methods

### Study Site

This study was conducted at a field site belonging to Bangladesh Rice Research Institute (24°0′0″N, 90°25′48″E), which is about 36 km north of Dhaka, the capital of Bangladesh. The site, which is located in a rice-growing area, has been used mainly for insect pest monitoring and forecasting since 1980.

### Data Collection

**Figure 1:**
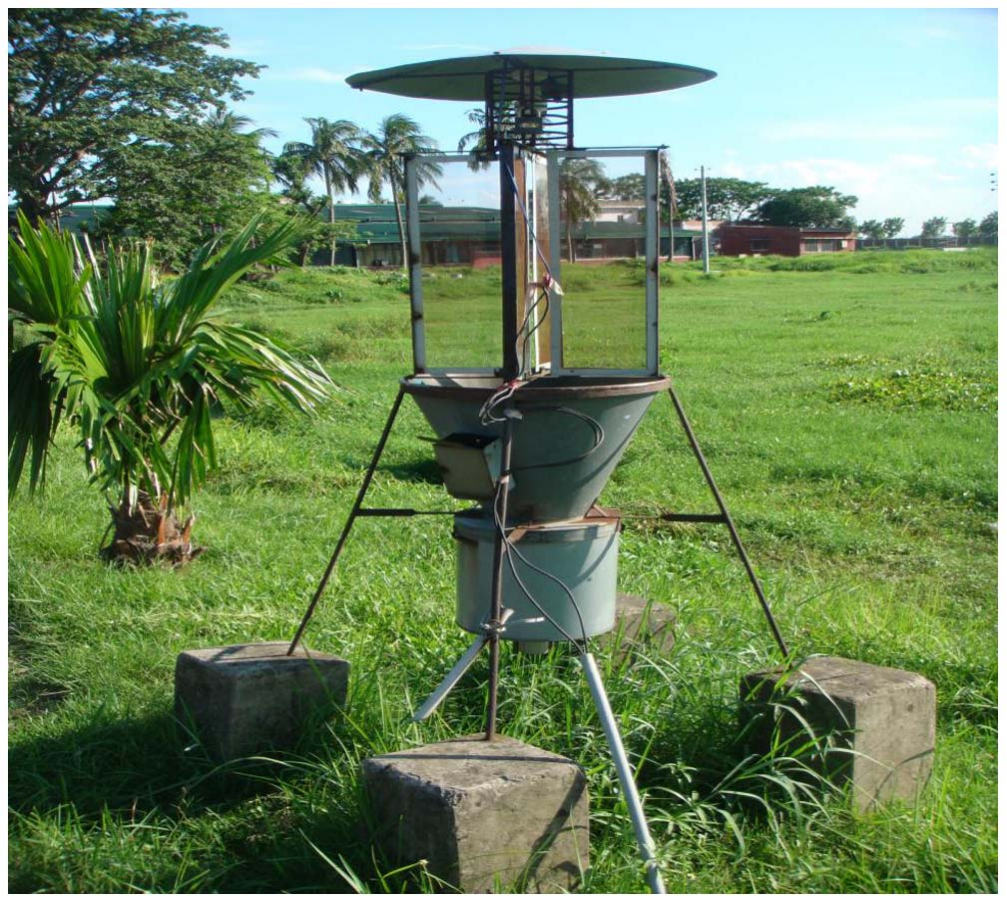
The light trap used to collect brown planthopper (BPH) in this study. It was placed adjacent to a rice field belonging to Bangladesh Rice Research Institute (BRRI).

The BPH population was monitored daily by a light trap (Fig. 1) which was placed close to rice fields. The trap used a 100-watt tungsten light bulb positioned at 2 m height. Lighting hours were set for 12 h (from evening to dawn) and a strip with dichlorvos (or Vapona) was put in each collecting cage to kill the insects when caught. A steel drum was placed below the cages to collect all insects including minute ones and to allow for their easy removal from the cages. The bulb and Vapona were changed when needed. The insects in the cages were removed every morning and preserved temporarily in paper bags dated daily before processing. Trapped insects were identified, counted and deposited in the Entomology Division of Bangladesh Rice Research Institute. There were no other lights near the trap. Monthly BPH catches from January to December from 1998 through 2007 were then calculated and used in this study (Appendix to manuscriptS).

### Climate Data Source

Climate variables (daily maximum and minimum temperatures, and precipitation) covering the same interval and study area were provided by the Plant Physiology Division of Bangladesh Rice Research Institute (www.brri.gov.bd).

### Statistical Analysis

#### Trends in climate and abundance of BPH from 1998 through 2007

We analyzed whether the climate data showed any long-term trends with respect to monthly temperatures and precipitation. For this purpose, we applied three climatic variables for every month through the 10 years period: *MinTemp* and *MaxTemp* which represent the average of the daily minimum and maximum temperatures during a month, and *Rain* which is the average amount of rain falling per day during the month. The three dependent variables were analyzed by a general linear model (PROC GLM in SAS [30]) with *Month* as a classification variable and *Year* (number of years since 1998) as a quantitative variable. The model also included the interactions between *Month* and *Year* to test whether trends differed among months. In the following, this model is termed the “null-model”. Overall, the model included 12 month parameters, 1 year parameter, and 12 Month X Year interaction terms, to a total degree of freedom of 23.

The monthly variation in BPH was analyzed in the same way as the climate variables, using the dependent variable *N* calculated as the average number of adult BPH caught per day during a month. Prior to the analyses we checked the dependent variables for normality and variance homogeneity. Variables that did not meet these requirements were transformed with the appropriate transformation obtained by means of Taylor’s power law [31].

#### Effect of weather variables on the abundance of BPH

The null-model predicting the expected number of BPH by means of the independent variables *Month* and *Year* and their interactions was expanded to include temperature and rainfall in order to investigate how much of the residual variance that could be accounted for by taking the actual weather in the month into account. The null-model expanded with the quantitative climate variables (called *Temp* and *Rain*) is termed the “full climatic model”. To avoid collinearity between the independent variables, minimum and maximum temperatures were not used in the same model because they tend to be positively correlated.

The full climatic model included first and second order terms of *Temp* and *Rain* plus all interaction terms between these independent variables. *Temp* could be either *MinTemp* or *MaxTemp*. Variables were transformed if needed to meet the criteria of normality and variance homogeneity. Analyses were conducted by means of PROC GLMSELECT in SAS [30] using a backwards elimination procedure based on Akaike’s corrected information criterion (AICC). The model with the lowest AICC was regarded as the best.

#### Predicted effects of climate change on BPH population

We used the general linear model identified above to simulate what will happen to the BPH if climate change results in higher temperatures in combination with a changing pattern of rain. We applied the following scenarios: Daily average temperatures were assumed to increase with 0°C, 1°C or 2°C and daily precipitation was either increased or decreased with 10%. The relative change in abundance of BPH for each month was calculated as R = 100(N−N_0_)/N_0_%, where *N* is the predicted catch of BPH for a given scenario and *N*_0_ is the corresponding value in the *status quo* scenario (i.e. the scenario with no climate change).

## Results and Discussion

**Figure 2:**
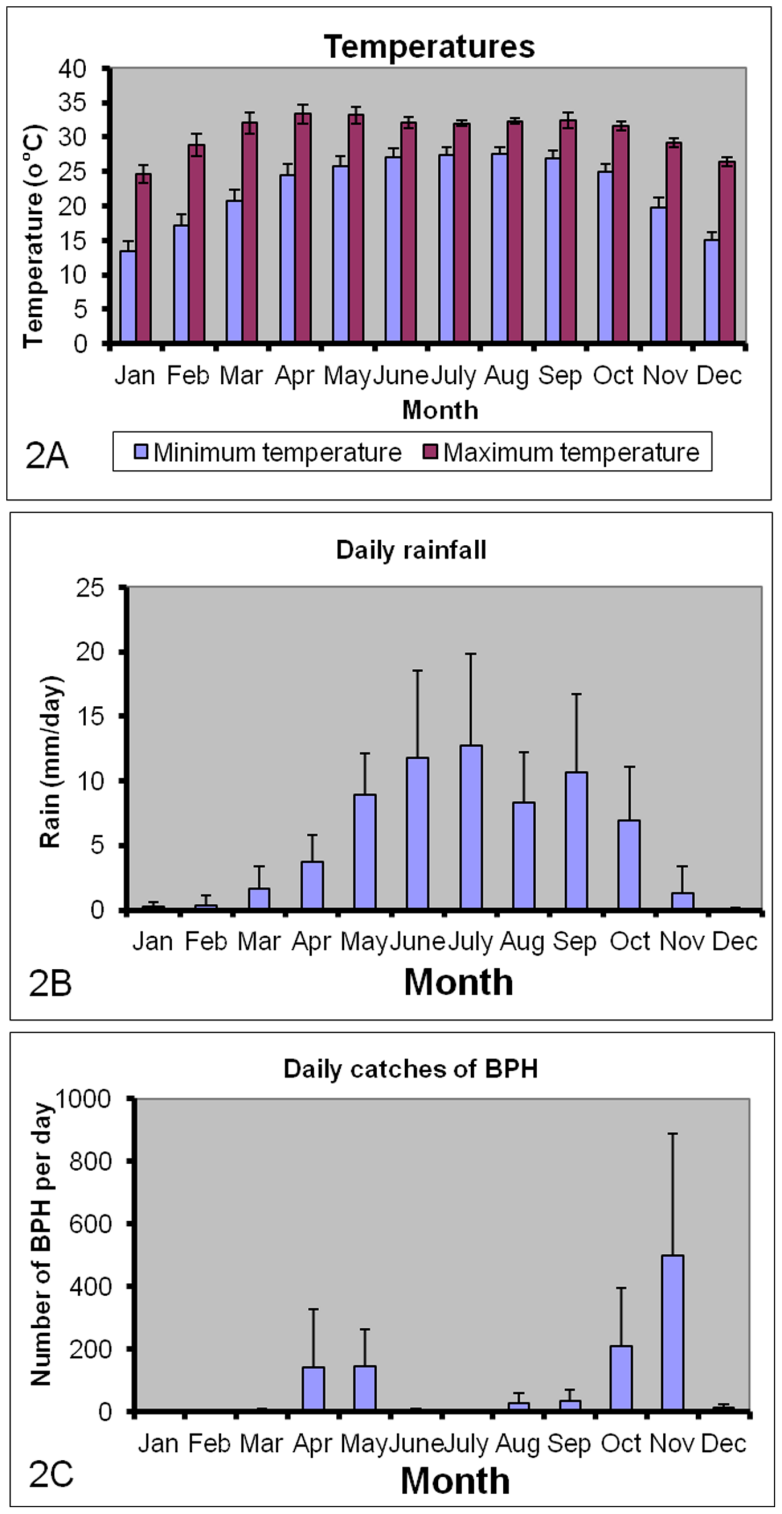
Monthly averages (±SD) of (A) Minimum and maximum temperatures; (B) daily rainfall; and (C) daily catches of Brown Planthoppers (BPH). Data from Bangladesh Rice Research Institute (BRRI).

The period from December to February is the cold and dry season with low abundance of planthoppers (Fig. 2A–C). Insect pests often respond rapidly and dramatically to changes in climatic conditions affecting development (such as sudden precipitation and extreme temperatures), leading to large temporal variations in insect pest populations [32].

### Trends in Climate and Abundance of BPH from 1998 Through 2007

**Figure 3:**
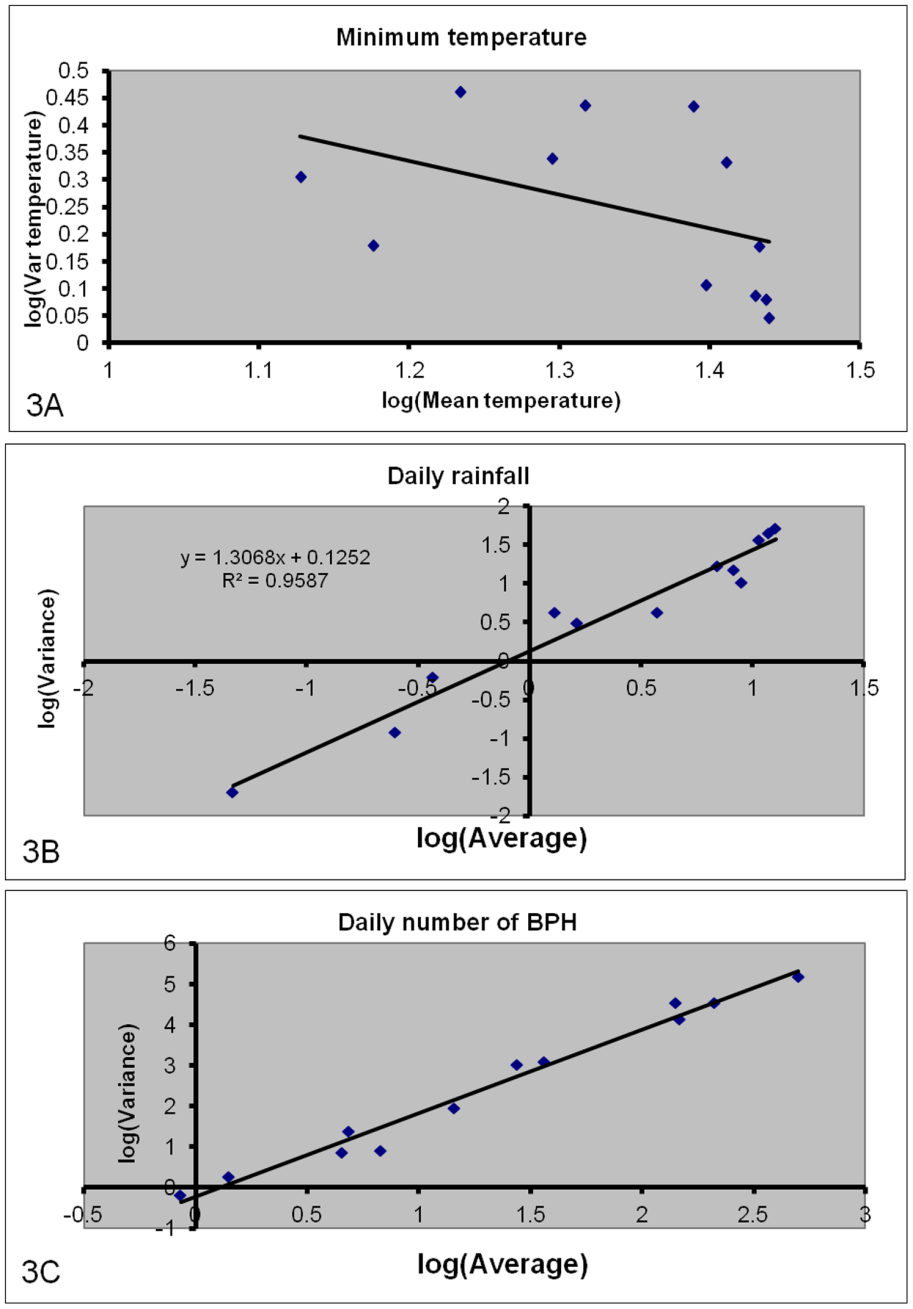
Log (variance) plotted against log(average) for (a) daily rainfall and (b) daily catches of BPH adults. Each dot represents a month. The straight line for daily rainfall is described by *y = *1.3068*x*+0.1252 (*R* ^2^ = 0.9587) and for daily catches of BPH by *y = *2.0512*x*–0.2258 (*R* ^2^ = 0.9793).

Figure 2 indicates that standard deviations of rainfall and BPH tend to increase with the average. We therefore applied Taylor’s power law [31] to identify the transformation of data that best stabilizes the variance. The appropriate transformations were obtained as 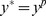 where *p* = 1−*b*/2, *b* is the slope of the regression line fitted to the empirical values of log(variance) plotted against log(average) from each of the 12 months (Fig. 3). As *p* for daily rainfall is close to one third, the cubic root transformation was used to transform daily rainfall values into *R* = *Rain* ^1/3^. For daily counts of PBH, *b* is close to 2, which means that this variable should be log transformed. As *N* in two cases was 0, we used the log(*N*+1) transformation. There was no need of transforming temperature values.

The model consisting of *Year, Month* and the interaction between them explained 97.2% of the variation in minimum temperatures, 89.0% in maximum temperatures and 76.8% in the transformed values of daily precipitation. However, for all three variables, neither *Year* nor the interactions between *Year* and *Month* were significant (*P>*0.05). There was a considerable monthly variation in both temperatures and rainfall, but the analysis did not indicate that this pattern has changed over the 10-years period.

With respect to a trend in BPH abundance, the null-model explained 84.8% of the variation in y* = log(*N*+1) (*F*_23,119_ = 23.21; *P<*0.0001). There was no overall trend over years (*P = *0.2468), whereas both *Month* and the interactions between *Month* and *Year* were significant (*Month*: *P<*0.0001; Year*Month: *P = *0.0020). Only April showed a significant trend in BPH over the 10 years with a slope of 0.1540 per year (*P*<0.0057). *Month* alone explained 79.4% of the total variation in log (*N*+1). As we could not demonstrate that the climate had changed, the most likely explanation for the increased abundance of BPH in April is that this month coincides with the growing and harvesting period of Boro rice, and that areas planted with Boro rice have expanded during the recent years [33]–[34].

### Effect of Weather Variables on the Abundance of BPH

When the null-model used above to predict the abundance of BPH was extended by including climatic variables, *MinTemp* had a higher predictive power than *MaxTemp*. We therefore used *MinTemp* to represent temperature in the following analyses. The full climatic model explained 86.3% of the variation in the observed values of log(*N*+1) (*F*_31,88_ = 17.9; *P*<0.0001). The AICC of the null-model (i.e. the model without climate variables) was −94.80. The climate-driven model with the lowest value of AICC (−107.12) contained the terms *Month, T, T* ^2^, *R, R* ^2^, and *T* ^2^ *R* ^2^, where *T* is the monthly minimum temperature (i.e. *T = MinTemp*) and. *R = Rain* ^1/3^. This reduced version of the full model (called the best climatic model) explained 83.6% of the variation in log(*N*+1) (*F*_16,103_ = 32.8; *P<*0.0001). The parameters associated with the model’s variables are shown in Table 1.

**Table 1:** Parameter values of the model that best describes the observed values of log(*N*+1), where *N* is the average number of BPH caught per day during a month.

Parameter	Estimate	Standard Error	t Value	*P*
**January**	−2.3351	1.5228	−1.53	0.1282
**February**	−2.3138	1.7269	−1.34	0.1832
**March**	−1.6957	1.8130	−0.94	0.3518
**April**	−0.2333	1.8110	−0.13	0.8978
**May**	0.1209	1.7931	0.07	0.9464
**June**	−1.0147	1.7756	−0.57	0.5689
**July**	−1.2602	1.7753	−0.71	0.4794
**August**	−0.6212	1.7711	−0.35	0.7265
**September**	−0.5186	1.7802	−0.29	0.7714
**October**	0.2358	1.8074	0.13	0.8964
**November**	0.0983	1.8015	0.05	0.9566
**December**	−1.4999	1.6344	−0.92	0.3609
***T***	0.3528	0.1767	2.00	0.0485
***T*** **^2^**	−0.0120	0.0044	−2.69	0.0083
***R***	0.7169	0.2094	3.42	0.0009
***R*** **^2^**	−0.9471	0.2213	−4.28	<.0001
***T*** **^2^** ***R*** **^2^**	0.00119	0.00029	4.09	<.0001

*T* is the temperature and *R* the cubic root of the daily rainfall in month *i.* The predicted value of log(*N*+1) for month *i* is obtained as 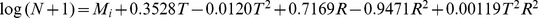 where *M_i_* is the parameter associated with month *i*. *P* is the probability that the true parameter value is equal to 0.

**Figure 4:**
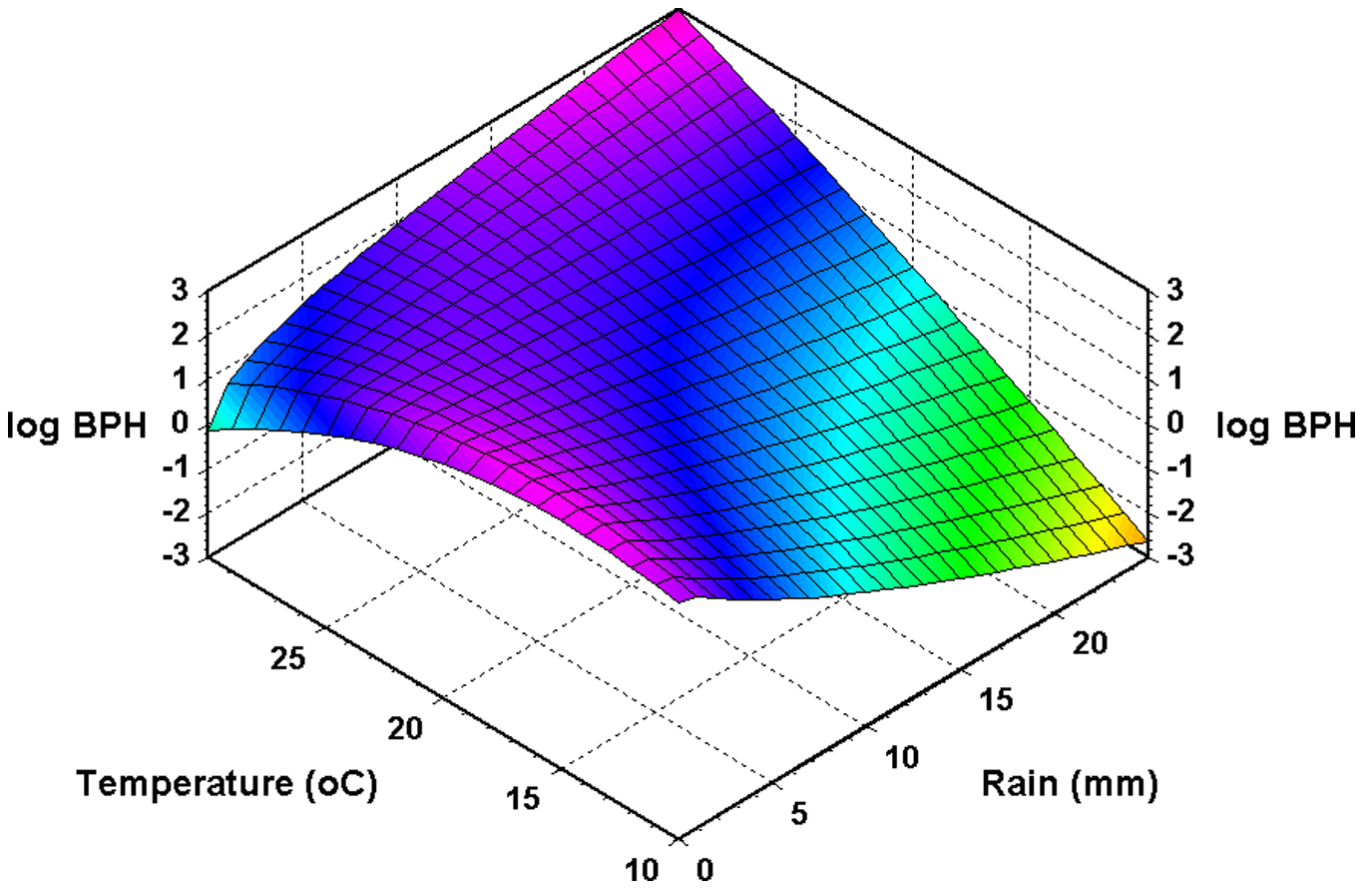
The effects of minimum temperature and rainfall on the predicted abundance of BPH (log(*N*+1)) (see Table 1 for further explanation).

Though the best climatic model explained slightly less of the variation than the null-model did (83.6% *vs* 84.8%), the former has 7 parameters fewer than the null-model. However, the main advantage of the climate-driven model is that it provides a tool for analyzing how temperature and rain influence the dynamics of BPH. Thus, the model’s parameters indicate that increasing temperatures will have a positive influence on BPH at low temperatures but a negative influence at high temperatures. Low levels of precipitation (e.g. <5 mm) will benefit BPH as long as the minimum temperature is below *ca* 23°C. Above this threshold, more rain will increase BPH. Figure 4 shows the predicted combined effects of temperature and rainfall when added to the monthly value of log(*N*+1).

### Predicted Effects of Climate Change on BPH Population

**Figure 5:**
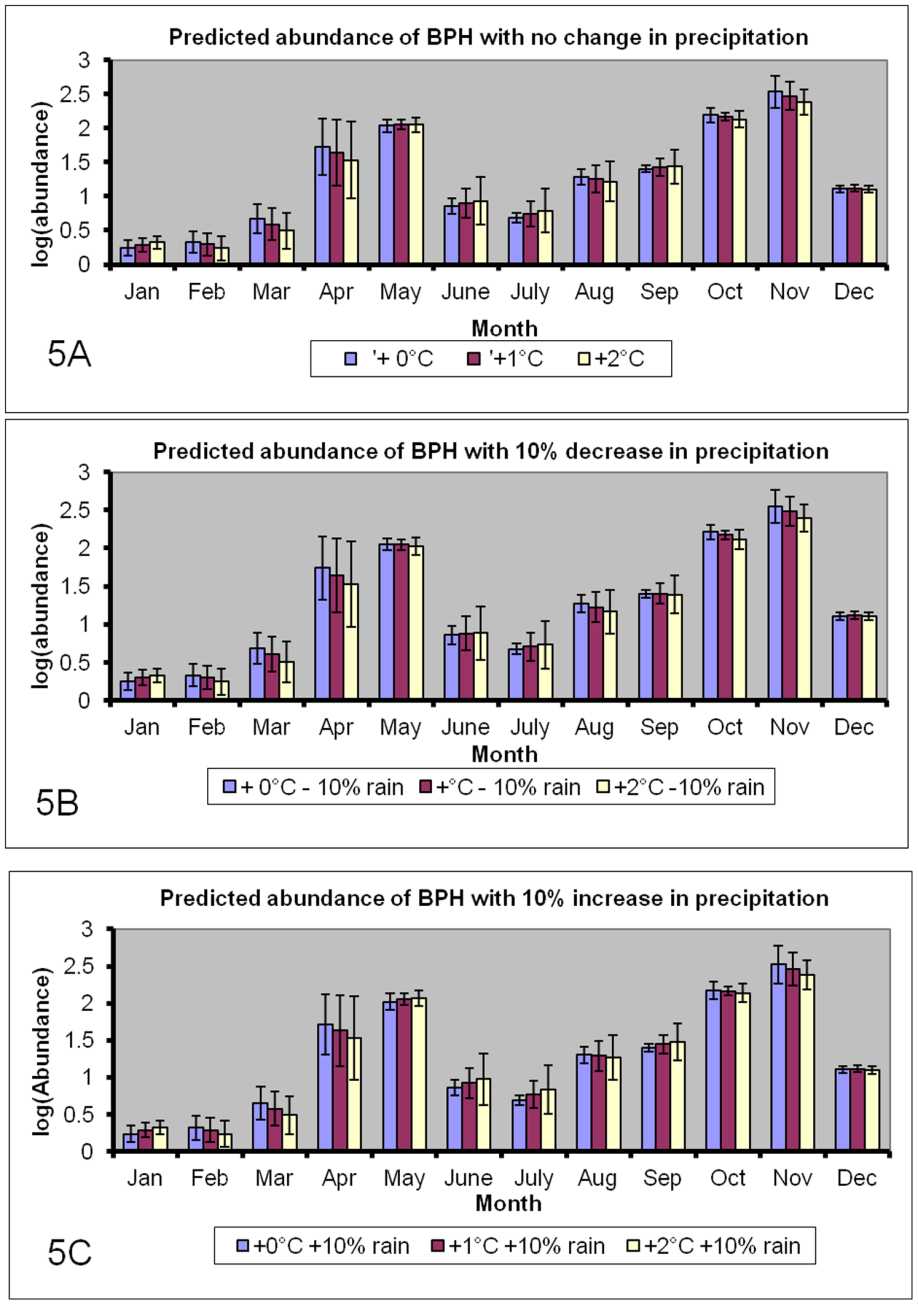
The predicted daily catches of adult BPH (±SD) if minimum temperature increases with either 0°C, 1°C or 2°C, and daily precipitation either decreases or increases with 10%.

**Figure 6:**
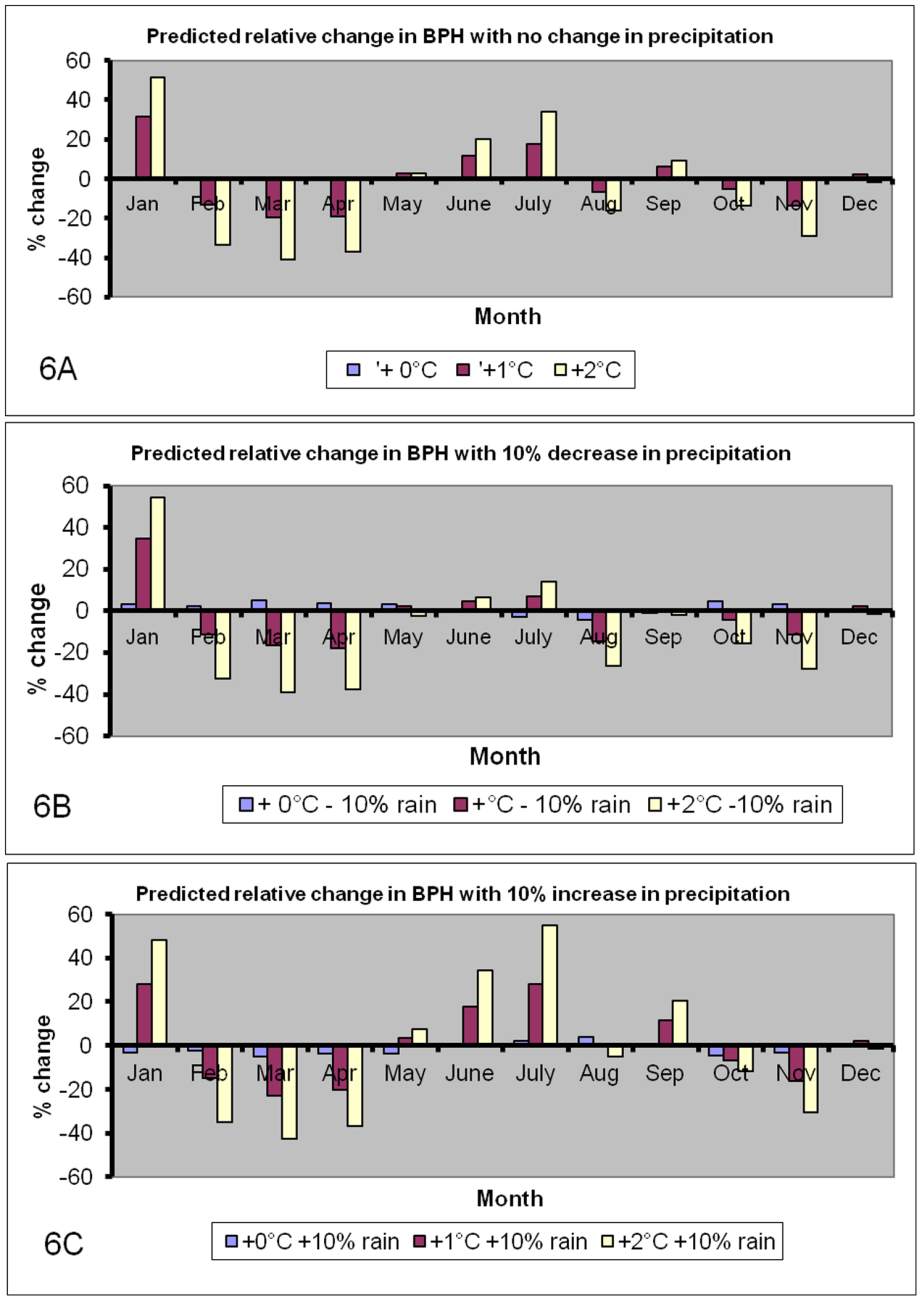
The predicted relative changes in daily catches of BPH under the various scenarios.

**Figure 7:**
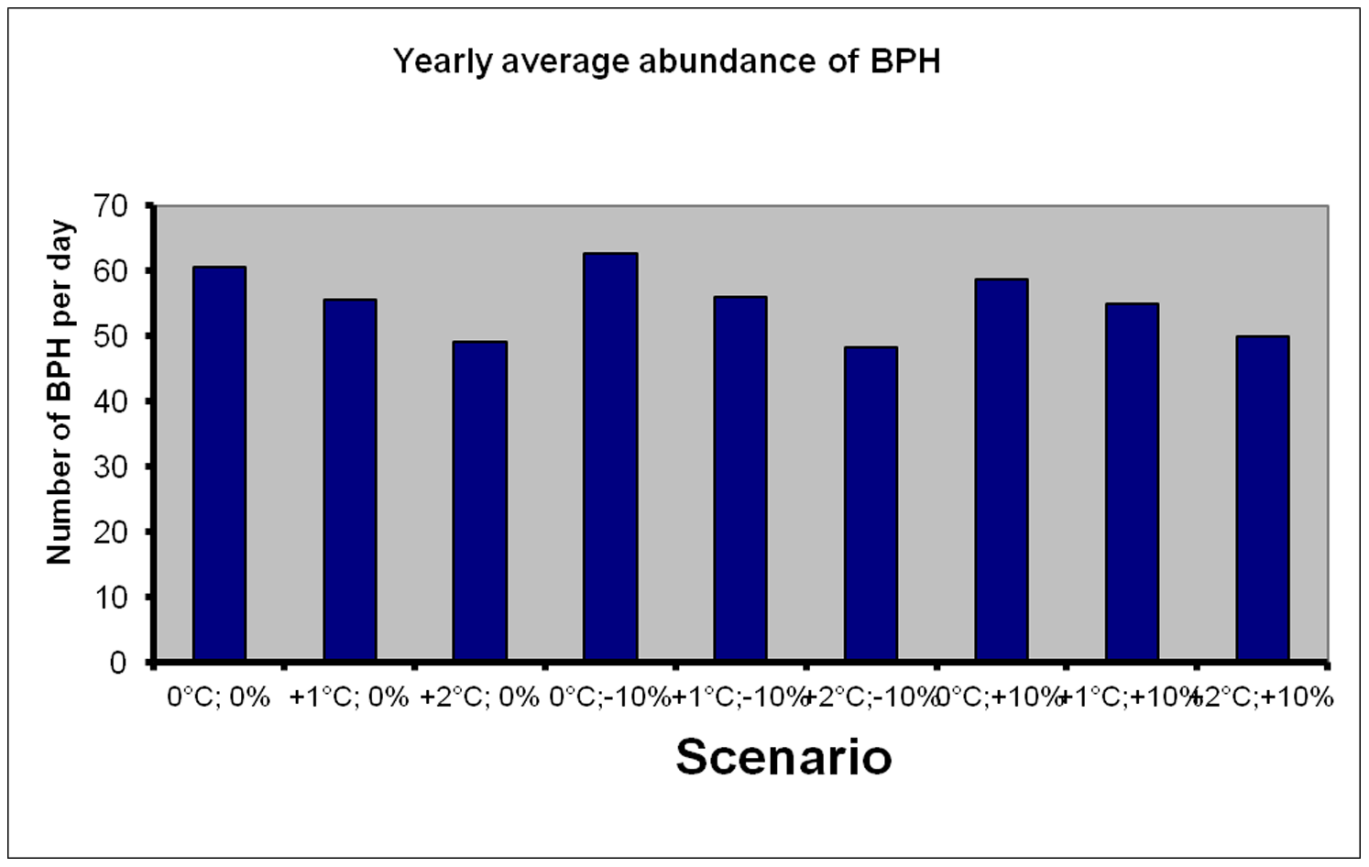
The predicted mean daily catches of BPH averaged over a year if daily minimum temperature changes with either 0°C, +1°C or +2°C, and if daily rainfall changes with either 0%, −**10% or +10%.**

Figure 5 shows the predicted monthly abundances of BPH under the various scenarios while Figure 6 shows the predicted relative changes in BPH abundance. The model predicts that BPH will become more abundant in January and during June-July, in particular if higher temperatures are associated with more precipitation. As these months are usually characterized by low abundances of BPH, the economic consequences are likely to be negligible. On the other hand, climate change is expected to decrease abundance of BPH during the spring and autumn seasons. These changes in seasonal patterns seem to benefit Boro rice crops (April–May), whereas rice grown in the Aus (July–August) and Aman (November–December) seasons will become more exposed to BPH. Overall, the daily mean number of BPH on a yearly basis is predicted to decline with an increase in temperature (Fig. 7). The only scenario that may increase BPH is the one where precipitation declines without a concurrent increase in temperature.

Even though our model indicates that BPH should become less abundant in Bangladesh if climate change causes an increase in the mean temperatures, it does not imply that this will be the case everywhere. Areas characterized by climatic conditions similar to those prevalent in Bangladesh during January (cool and dry) or July (hot and wet) seems most vulnerable to a changing climate. Using the model in combination with GIS-based climate information could assist in pointing out regions where future rice production is jeopardized.

The results confirm that the combined effect of temperature and rainfall is complex [35]–[36]. Global warming may increase evaporation and precipitation with 10 to 15% because a warmer atmosphere holds more moisture [24]–[25]. In warm areas this is expected to increase populations of BPH. In some areas, however, global warming is predicted to result in a drier climate. Thus, Dai [37] suggests severe and widespread droughts in the next 30–90 years over many land areas. According to the model, this may also exaggerate problems with BPH, but only in areas where minimum temperatures stay below *ca* 23°C.

Climate change, and particularly global warming, may have a dramatic impact on pest insect species [38]. Many authors predict that climate change will have a number of effects on insects: sweeping shifts in herbivory rates; altered distribution and outbreak frequency of key insect pests; unpredictably altered relationships with natural enemies; and a general decrease in biodiversity [39]–[41]. The findings of the present study indicate that the reported BPH outbreak in rice fields occurring during the warm seasons might be attributed to global warming [42]. However, the model also shows that temperatures exceeding 23°C in a dry climate may have an inhibiting effect on population density, implying that the consequences of global warming on planthoppers are ambiguous. Thus, their long-term abundance may increase during the cold season and decrease during the hot season, depending on how the ambient temperatures change relative to the threshold value of *ca* 23°C. Planthopper outbreaks were observed in the boro rice growing season i.e. the dry season [43] and significant BPH outbreaks were recorded during the dry season in the early 1990s in the Vientiane Plain [44] along with higher temperatures. The population subsequently declined in January due to low temperatures and peaked again in March and April 2008 during the dry season [45] when temperatures were high. These weather conditions seem to promote BPH outbreaks, which is in accordance with the predictions based on our model, because minimum temperatures in the area during the dry season rarely exceed 23°C [12].

Wide-scale outbreaks of BPH in tropical Asian rice in the 1970s and 1980s [46]–[47] were attributed to the insecticidal destruction of natural enemies [48]. One of the main causes of the recent series of outbreaks in Thailand and Indonesia was also insecticide misuse [49]. Although insecticide applications to rice do not always trigger BPH outbreaks [46], they often disrupt the actions of BPH’s natural enemies either by their direct killing action or by disrupting food chains [50]. Spraying of insecticide in rice field for controlling other pests destroys essential ecosystem services that regulate invading planthopper adults, thus increasing the risk of hopperburn on the farm [11]. Sublethal insecticide applications could theoretically increase BPH’s capacity for migration because the planthoppers would acquire more fat and sugar, which provide fuel for flight, when feeding on insecticide-treated rice plants than when feeding on untreated plants [49]. The applications of sublethal doses of certain insecticides could enhance both BPH’s reproductive and migratory capacity and theoretically increase the threat of BPH outbreaks even if the insecticides do not harm natural enemies [51].

It is tempting to conclude that human-controlled inputs such as insecticides or insecticides in combination with nitrogen fertilizer, for example, have been totally responsible for the synchronous BPH outbreaks in tropical rice observed across many areas of Asia shortly after the beginning of the green revolution and again more recently [49], but there is a widespread concern that outbreaks of herbivorous insects will increase in frequency and severity as a consequence of global warming. These predictions, however, are untested and still very uncertain.

In an environment conducive to more rapid BPH increases, natural enemies and BPH-resistant cultivars would be expected to have less effect in regulating the density of BPH, and pesticides harming natural enemies would therefore be expected to have an above-average negative impact [49]. Kiritani [27] also reported that every 1°C rise from around *X* = 4°C would result in a decrease in winter mortality of about 16.5% based on a regression model linking winter mortality (*Y*) to the mean temperature in January (*X*).

*N. lugens* is a long-range migrant species that immigrates into Japanese paddy fields from mainland China in early summer [52]. Our study does not consider immigration of BPH into the study area because this phenomenon has not yet been observed in Bangladesh. Moreover, we accentuate that our model is a simple measure of the importance of climate compared to all other factors (such as pesticides, natural enemies etc). It does not focus on the overall causes for pest outbreaks, nor does it independently attribute pest trends to the many technological, climatic and biotic factors that may affect such trends. However, it discloses a pragmatic approach to assessing the combined importance of climate variables with special reference to evaluating the possible impact of climate change on pest outbreaks.

Long time series like ours provide a good basis for linking pest outbreaks to climate change and to separate the influence of other factors, such as pesticides, from that of climate. Our study indicates that climate change may have contributed to the recent destructive outbreaks of planthoppers in rice crops [53]. However, more research is needed to predict more accurately the long-term consequences of a changing climate, and especially how such changes will affect pest dynamics at both regional and local scales.

## Conclusion

Data collected from 1998 through 2007 did not indicate that temperature and precipitation patterns have changed significantly during this 10-years period. Therefore, we attribute the significant increase in BPH during April to changes in agricultural practice rather than climate change. However, the statistical models demonstrate that temperature and rainfall are driving variables for the dynamics of BPH, so any changes in these factors due to climate change are likely to affect the outbreak patterns of BPH. We tested this by simulating different climatic scenarios and found a quite complex picture depending on the severity and direction of the expected climatic changes. Thus, our study emphasizes the importance of taking future climate into consideration when deciding which, where and when the different rice cultivars should be planted and harvested so as to minimize crop losses due to pests.

Given the challenge of linking pest impacts and directional climate change for well-studied agricultural, maricultural (cultivation of marine organisms in their natural habitats, usually for commercial purposes), and human diseases, it is not yet possible to predict the consequences for biodiversity. Very few empirical studies directly explore the relationship between climate and rice pest problems. Even fewer explore interactions between temperature and various components of an insect’s life cycle [54]–[56]. We therefore identify three priorities for research to improve our ability to predict impacts of climate change on rice pests:

1. *Collect baseline data on insects of both major and minor importance*: Baseline data are critical to predict changes in a warmer climate, but such data are rarely collected for rice ecosystems. Monitoring programs for the prevalence and severity of pests and their population- and community-level impacts must be implemented for a wider range of natural systems.
2. *Separate the effects of multiple climate variables on insects*: To predict accurately future responses to climate change, we must quantify the direct and synergistic effects of multiple climate variables, such as temperature and precipitation, on insects. Laboratory and/or field experiments are crucial for studying the specific effects of these variables [57].
3. *Forecast epidemics*: Forecasting models using climate variables can effectively predict outbreaks for some crops and pests. Crop disease programs have long been in effect as, e.g., rice blast (*Pyricularia oryzae*) models based on temperature and moisture forecasts when an epidemic will start and when to apply fungicide for optimal control [58]. Such forecasting programs are also in development for human diseases with climate sensitivity, such as Rift Valley fever, which is associated with warm El Niño events of high rainfall [58]–[59], and cholera, which is predictable from sea surface temperature associations with El Niño [59]. Similar forecasting models will be very useful for predicting epidemic outbreaks of insect pests in rice and we hope that the present paper will contribute to achieving this goal.

## Supporting Information

Appendix S1 **Data used in this study.**
(XLS)
